# Vaccines as antimicrobial resistance control tools: evidence from pneumococcal conjugate vaccines in South Africa

**DOI:** 10.11604/pamj.supp.2025.51.1.48165

**Published:** 2025-06-13

**Authors:** Chinwe Iwu-Jaja, Akhona Victress Mazingisa, Anelisa Jaca, Chidozie Declan Iwu, Charles Shey Wiysonge

**Affiliations:** 1 World Health Organization, Regional Office for Africa, Brazzaville, Congo,; 2 Department of Community Health Studies, Faculty of Health Sciences, Durban University of Technology, Durban 4001, South Africa,; 3 Cochrane South Africa, South African Medical Research Council, Cape Town, South Africa,; 4 School of Health Systems and Public Health, Faculty of Health Sciences, University of Pretoria, Pretoria, South Africa

**Keywords:** Antimicrobial resistance, impact of vaccines on antimicrobial resistance, vaccine uptake, pneumococcal conjugate vaccines, immunisation, Africa

## Abstract

The Expanded Programme on Immunisation demonstrated remarkable success for 50 years, accounting for 52% reduction in infant mortality in Africa. As antimicrobial resistance (AMR) continues to be a threat in Africa, responsible for 250,000 deaths in WHO African region in 2019, vaccines offer proven solutions. Studies from South Africa demonstrate that pneumococcal conjugate vaccines significantly reduced drug-resistant pneumococcal infections, with 67-96% reductions in resistant strains. Increasing immunisation coverage in Africa, reduces disease burden while providing a cost-effective strategy to combat antimicrobial resistance.

## Brief

The Expanded Programme on Immunisation (EPI), launched 50 years ago, has demonstrated unprecedented success in reducing mortality, averting 154 million deaths globally and accounting for 52% of the decline in infant mortality in the African region [1]. This remarkable achievement underscores the proven track record of vaccines as one of the most effective public health interventions in history. Antimicrobial resistance threatens to undermine decades of progress in fighting infectious diseases in Africa. Bacterial AMR directly caused 250,000 deaths in 2019 [2]. By 2050, bacterial AMR could result in up to 1.91 million annual deaths globally, disproportionately affecting the WHO African region [2]. Healthcare systems face mounting costs treating resistant infections, with longer hospital stays and higher mortality rates [3], particularly concerning in resource-limited settings where alternative antibiotics may be unavailable.

Building on EPI’s proven success, vaccines offer a complementary approach to addressing AMR, by preventing infections and reducing antibiotic use, thereby decreasing selection pressure for resistant organisms [4,5]. While various vaccines have demonstrated potential in addressing antimicrobial resistance (AMR), pneumococcal conjugate vaccines (PCVs) represent the most extensively studied intervention with the most robust available empirical evidence in Africa [6,7].

Streptococcus pneumoniae is a leading cause of pneumonia, meningitis, and bloodstream infections and deaths globally, with Africa bearing a disproportionate burden. PCVs have led to significant reduction of these diseases, particularly among children under five years [8]. More recently, their role in reducing antibiotic-resistant strains has gained attention. Given the pressing need to highlight the role of vaccines in curbing AMR for policy makers and stakeholders in immunisation and AMR space, this paper summarises the available evidence on the impact of PCV on AMR in Africa.

**Figure 1:**
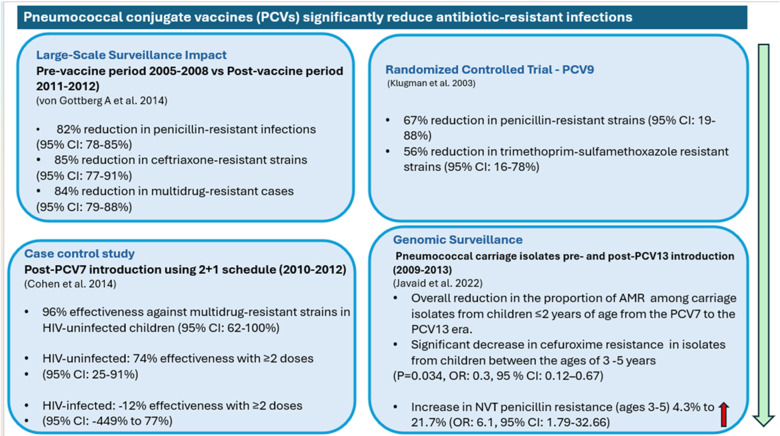
impact of pneumococcal conjugate vaccines on antibiotic resistance

Evidence from South Africa demonstrates PCV’s impact on AMR through four key studies. These include a randomised controlled trial (RCT) [9], a case-control study [10], a clinical surveillance study [11], and a genomic surveillance study [12]. The RCT which was a study which assessed the efficacy of PCV9, demonstrated a 67% reduction in penicillin-resistant strains (95% CI: 19-88%) and 56% reduction in trimethoprim-sulfamethoxazole resistant strains (95% CI: 16-78%) [9]. The case-control study following PCV7 introduction (2010-2012) showed 96% effectiveness against multidrug-resistant strains in HIV-uninfected children (95% CI: 62-100%) [10]. The clinical surveillance study which compared pre-vaccine (2005-2008) and post-vaccine periods (2011-2012) showed significant reductions in resistant infections: 82% decrease in penicillin-resistant infections (95% CI: 78-85%), 85% reduction in ceftriaxone-resistant strains (95% CI: 77-91%), and 84% decline in multidrug-resistant cases of pneumococcal disease (95% CI: 79-88%) [11]. Lastly, the genomic surveillance revealed significant changes in resistance patterns between PCV7 and PCV13 eras [12]. Among children ≤2 years, substantial reductions were observed for several important antibiotic classes. Most notably, resistance to penicillin decreased from 55.5% to 41.8%, while cephalosporin resistance showed similar declines (cefuroxime: 9.9% to 3.2%; ceftriaxone: 6.4% to 1.5%). Significant reduction in cefuroxime resistance was also observed in children aged 3-5 years (OR: 0.3, 95% CI: 0.12-0.67, P=0.034). Furthermore, vaccine-type strains (those targeted by the vaccine formulation) showed stronger association with reduced resistance compared to non-vaccine types (strains that aren’t targeted by the vaccine formulation). However, among non-vaccine types, penicillin resistance significantly increased in children aged 3-5 years (4.3% to 21.7%, OR: 6.1, 95% CI: 1.79-32.66, P=0.02) [12]. These findings are summarised in Figure 1.

The robust evidence from South Africa demonstrates that PCV significantly reduce antibiotic-resistant infections, providing the real-world evidence of vaccines’ potential in combating AMR. While emerging data from other vaccines, such as respiratory syncytial virus (RSV) and typhoid conjugate vaccines, show promise in reducing antibiotic use in Africa, the compelling evidence from PCV serves as a compelling example of the expanded value of vaccines. This evidence supports a paradigm shift in how we value vaccination programmes: not just as disease prevention tools, but as crucial and cost-effective interventions in addressing the global challenge of AMR.

For policy makers, these findings provide additional rationale for strengthening existing immunisation programmes and justify investment in vaccination as one of the core strategies for AMR control. Beyond preventing deaths, reduced AMR infections decrease healthcare costs through shorter hospital stays and reduced need for expensive second-line antibiotics. Investing in initiatives to improve vaccine uptake and coverage therefore offers a dual benefit: preventing infectious diseases while simultaneously tackling AMR. Priority actions should include integrating AMR impact assessment into immunisation programme evaluations alongside existing coverage improvement strategies. As novel vaccines enter the pipeline, their potential impact on AMR should be considered in policy decisions. In an era of dwindling global health financing, this dual-impact approach maximises the value of every health dollar spent on vaccination programmes.

In conclusion, as Africa celebrates 50 years of the Expanded Programme on Immunisation [13,14], the demonstrated success of PCV in reducing antibiotic-resistant infections exemplifies how immunisation programmes can address critical public health challenges like AMR while providing compelling evidence for strengthening vaccination programmes as a key strategy. Further research from other African countries and on additional vaccines is needed to build a more comprehensive evidence base.
